# The Resting Position of Anopheles

**Published:** 1900-10-27

**Authors:** 


### THE RESTING POSITION OF ANOPHELES.

It is always well to correct error at once, for the makers of medical books have a bad habit of copying one from another, so that an error once set agoing may pass on through many generations of handbooks and encyclopædias. Major Donald Ross and Mr. A. E. Austen in the report of their expedition to Sierra Leone in 1899 laid great stress on the position assumed by mosquitoes when at rest, as a means of discriminating between the malaria-bearing mosquitoes of the genus *Anopheles* and those of the genus *Culex*, the latter of which so far seem to take no part in the dissemination of human malaria. They said that the *Anopheles* always, when at rest, sat in a characteristic attitude, their bodies being maintained at an angle of at least 45° to the surface on which they settled. Frequently, they said, the tips of the palpi were in contact with the wall on which the insect was resting, and the body was so much elevated that it was practically at right angles to the wall, and the insect appeared as if it were standing upon its head, and it was held that, owing to this extremely characteristic resting attitude of *Anopheles*, it would be impossible for anyone who had once seen a specimen at rest to mistake it for a *Culex*, for the latter when at rest allows its body to hang down vertically.

Now Dr. Sambon and Dr. Low [1] have been passing the summer on the Campagna, and they directly traverse this statement as to the attitude of these insects. They say that they have observed thousands of *Anopheles*, but none of them ever assumed the position described by the members of the Liverpool Malarial Mission, and they hold that although the resting position may be a means of distinguishing between certain species, it is not a criterion by which *Anopheles* can be distinguished from *Culex*, which was the point insisted on, a point which has already been copied into many text books.

[1] Brit. Med. Jour., Oct. 20.

